# Prognostic value of serum galactomannan in mixed ICU patients: a retrospective observational study

**DOI:** 10.1186/cc10642

**Published:** 2012-03-20

**Authors:** S Teering, A Verreth, W Verlinden, J Jacobs, S Pilate, M Peetermans, A Verrijcken, N Van Regenmortel, I De laet, K Schoonheydt, H Dits, M Van De Vyvere, M Malbrain

**Affiliations:** 1 ZNA Stuivenberg, Antwerp, Belgium

## Introduction

Little is known about galactomannan (GM) testing in mixed ICU patients that are often not neutropenic. The aim of this study was to look for the incidence and outcome of invasive aspergillosis (IA) in critically ill patients, to validate previous reported GM thresholds and to evaluate the prognostic value of GM.

## Methods

A retrospective study of 474 GM samples in 160 patients from 1 January 2003 to 1 February 2004. GM tests were ordered because of clinical suspicion of IA or on a regular basis in immune compromised patients. The number of samples per patient was 3 ± 2.6. Similarly to the EORTC criteria we defined 'proven IA' as those patients with positive tissue specimen, 'probable IA' as those with positive cultures, and 'possible IA' as those treated with antifungals (high clinical index of suspicion). The number of positive samples (GM >0.5 ng/ml) was 230 (48.5%). Patient characteristics: M/F ratio 1/1, age 64.5 ± 15.9, SAPS 45.5 ± 16.8, APACHE II 19.3 ± 8, SOFA 5.8 ± 3.5, mean days on ventilation 12.9 ± 8.7, mean CRP 10.4 ± 11.2 mg/dl.

## Results

In our study population 5% had proven IA, 5% probable IA, 17.5% possible IA and 72.5% had no IA. We could not identify a GM threshold for IA. The best threshold was GM >1.1 for identifying patients with IA (proven + probable + possible) with a specificity of 70.7% and negative predictive value of 76.6%. The ICU mortality was 41.9% and the hospital mortality was 58.1%. Patients who died in the ICU had higher APACHE, SAPS and SOFA scores (*P *< 0.0001), and had a significant increase in GM during their stay (0.27 ± 1.26 vs.-0.43 ± 1.7, *P *= 0.004). We observed higher mean GM values in nonsurvivors but this was not statistically significant. Patients who died in the hospital also showed a significant increase in GM during their stay (0.11 ± 1.55 vs. -0.48 ± 1.51, *P *= 0.017). There was a trend towards higher GM values in patients treated with piperacillin/tazobactam (*n *= 34) but this was not statistically significant. Neutropenic patients (*n *= 31) showed an increase in GM during their stay (0.32 ± 1.3 vs. -0.43 ± 1.7, *P *= 0.07). Patients on total parenteral nutrition (*n *= 125) had higher maximal GM levels (1.55 ± 1.94 vs. 0.88 ± 1.25, *P *= 0.058). Patients that were mechanically ventilated had significantly higher mean (*P *= 0.038) and maximal (*P *= 0.007) GM levels. The presence of IA was associated with 100% hospital mortality.

## Conclusion

The current GM threshold of 0.5 ng/ml does not allow one to discriminate between patients with and without IA. A threshold of 1.1 ng/ml had the best specificity and negative predictive value for IA. There seems to be a correlation between GM levels and total parenteral nutrition due to interference with the ELISA test.

